# FusionVAC22_01: a phase I clinical trial evaluating a DNAJB1-PRKACA fusion transcript-based peptide vaccine combined with immune checkpoint inhibition for fibrolamellar hepatocellular carcinoma and other tumor entities carrying the oncogenic driver fusion

**DOI:** 10.3389/fonc.2024.1367450

**Published:** 2024-03-28

**Authors:** Christopher Hackenbruch, Jens Bauer, Jonas S. Heitmann, Yacine Maringer, Annika Nelde, Monika Denk, Lisa Zieschang, Christine Kammer, Birgit Federmann, Susanne Jung, Peter Martus, Nisar P. Malek, Konstantin Nikolaou, Helmut R. Salih, Michael Bitzer, Juliane S. Walz

**Affiliations:** ^1^ Clinical Collaboration Unit Translational Immunology, German Cancer Consortium (DKTK), Department of Internal Medicine, University Hospital Tübingen, Tübingen, Germany; ^2^ Department of Peptide-based Immunotherapy, Institute of Immunology, University and University Hospital Tübingen, Tübingen, Germany; ^3^ Cluster of Excellence iFIT (EXC2180) “Image-Guided and Functionally Instructed Tumor Therapies”, University of Tübingen, Tübingen, Germany; ^4^ German Cancer Consortium (DKTK) and German Cancer Research Center (DKFZ), partner site Tübingen, Tübingen, Germany; ^5^ Institute for Medical Biometrics and Clinical Epidemiology, University Hospital Tübingen, Tübingen, Germany; ^6^ Department of Internal Medicine I, University Hospital Tübingen, Tübingen, Germany; ^7^ Center for Personalized Medicine, University of Tübingen, Tübingen, Germany; ^8^ The M3 Research Institute, University of Tübingen, Tübingen, Germany; ^9^ Department of Diagnostic and Interventional Radiology, University Hospital Tübingen, Tübingen, Germany

**Keywords:** FLC, FL-HCC, DNAJB1-PRKACA fusion transcript, neoepitope, peptide vaccination, immune checkpoint inhibition

## Abstract

The DNAJB1-PRKACA fusion transcript was identified as the oncogenic driver of tumor pathogenesis in fibrolamellar hepatocellular carcinoma (FL-HCC), also known as fibrolamellar carcinoma (FLC), as well as in other tumor entities, thus representing a broad target for novel treatment in multiple cancer entities. FL-HCC is a rare primary liver tumor with a 5-year survival rate of only 45%, which typically affects young patients with no underlying primary liver disease. Surgical resection is the only curative treatment option if no metastases are present at diagnosis. There is no standard of care for systemic therapy. Peptide-based vaccines represent a low side-effect approach relying on specific immune recognition of tumor-associated human leucocyte antigen (HLA) presented peptides. The induction (priming) of tumor-specific T-cell responses against neoepitopes derived from gene fusion transcripts by peptide-vaccination combined with expansion of the immune response and optimization of immune function within the tumor microenvironment achieved by immune-checkpoint-inhibition (ICI) has the potential to improve response rates and durability of responses in malignant diseases. The phase I clinical trial FusionVAC22_01 will enroll patients with FL-HCC or other cancer entities carrying the DNAJB1-PRKACA fusion transcript that are locally advanced or metastatic. Two doses of the DNAJB1-PRKACA fusion-based neoepitope vaccine Fusion-VAC-XS15 will be applied subcutaneously (s.c.) with a 4-week interval in combination with the anti-programmed cell death-ligand 1 (PD-L1) antibody atezolizumab starting at day 15 after the first vaccination. Anti-PD-L1 will be applied every 4 weeks until end of the 54-week treatment phase or until disease progression or other reason for study termination. Thereafter, patients will enter a 6 months follow-up period. The clinical trial reported here was approved by the Ethics Committee II of the University of Heidelberg (Medical faculty of Mannheim) and the Paul-Ehrlich-Institute (P-00540). Clinical trial results will be published in peer-reviewed journals.

**Trial registration numbers:**

EU CT Number: 2022-502869-17-01 and ClinicalTrials.gov Registry (NCT05937295).

## 1 Introduction

Fibrolamellar hepatocellular carcinoma (FL-HCC), also known as fibrolamellar carcinoma (FLC), is a rare primary liver malignancy with a 5-year survival of only 45%, which typically affects young patients (mean age ∼22 years) without any underlying primary liver disease (1, 2). FL-HCC is a rare but increasing cancer (3, 4). Surgical resection is the only curative treatment option, if no metastases are present at diagnosis (5, 6). As FL-HCC is often diagnosed in an advanced or metastasized stage, surgery is available for only a limited number of patients, and tumor recurrence after surgery is often observed (5, 7, 8). In selected patients, orthotopic liver transplantation is performed with similar overall survival (OS) compared to the survival of hepatocellular carcinoma (HCC) patients after orthotopic liver transplantation (9). Local ablative procedures like radiotherapy or interventional radiological measures, such as transcatheter chemoembolization, are used in multimodal treatment concepts for individual FL-HCC patients, but the effect of these treatment modalities on survival is still unclear (10–15). There is no standard of care regarding systemic therapy (3, 5, 15). FL-HCC is often treated with chemotherapy or targeted therapy regimes that are approved for the more frequent but distinct tumor entity HCC without substantial improvement of the long-term survival rates of FL-HCC patients (1–3, 5, 7–9, 14, 15). Therefore, attempts are made to study specific therapies for FL-HCC, with some of these, even though mostly not documented in prospective trials, showing some degree of efficacy, including (i) fluorouracil and interferon alfa-2b, in particular in combination with nivolumab, and (ii) sorafenib or lenvatinib especially in combinatorial treatment regimens, e.g. with gemcitabine/oxaliplatin (16–19) or combinatorial treatment with the BCL-XL targeting proteolysis targeting chimera (PROTAC) DT2216 in combination with irinotecan (20). Nevertheless, the limited success of available systemic therapies calls for the development of new treatment options for FL-HCC patients.

The DNAJB1-PRKACA fusion transcript was recently detected as the oncogenic driver of tumor pathogenesis in almost 100% of FL-HCC patients and thus represents an attractive target for the development of novel therapies for this devastating tumor disease (21–24). Furthermore, recent advances in genome sequence analysis (25, 26) enabled the identification of further cancer entities (e.g. oncocytic neoplasms of the pancreas and bile duct) that express the DNAJB1-PRKACA fusion transcript (27) giving the prospect of future off-the-shelf therapies targeting the DNAJB1-PRKACA fusion in multiple cancer entities.

Peptide-based vaccines represent a low side-effect approach relying on specific immune recognition of tumor-associated human leucocyte antigen (HLA) presented peptides. Several peptide vaccination studies have reported promising results in solid tumors (28–31) and hematologic malignancies (32–35) in terms of *in vivo* immunogenicity, however so far lacking broad clinical responses. In extensive preclinical work our group showed that the DNAJB1-PRKACA fusion transcript is a source for naturally-presented immunogenic neoepitopes and can be actively targeted by T-cell-based immunotherapy (36). First application of a DNAJB1-PRKACA fusion transcript-based peptide-vaccine adjuvanted with the Toll-like receptor (TLR) 1/2 agonist XS15 emulsified in Montanide ISA 51 VG to a FL-HCC patient was well tolerated and showed the induction of profound and long-lasting T-cell responses accompanied by long-term disease-free survival (36). Another important factor for the clinical effectiveness of peptide-based immunotherapy is the rational combination with other immunotherapies or cancer drugs. Immune-checkpoint-inhibition (ICI) has been used in the treatment of FL-HCC patients, and even though data on efficacy are still rare and mainly case reports exist, a modest benefit has been reported in a small analysis (19, 37–40). Various data have confirmed the rational combination of vaccination approaches with ICI and its therapeutic potential [reviewed in (41) (42–44)].

In the phase I clinical trial reported here, we evaluate the DNAJB1-PRKACA fusion transcript-based neoepitope vaccine Fusion-VAC-XS15 in combination with the anti-programmed cell death-ligand 1 (PD-L1) antibody atezolizumab (Tecentriq™) in patients with FL-HCC or other locally advanced or metastatic cancer entities carrying the DNAJB1-PRKACA fusion transcript.

## 2 Methods and analysis

### 2.1 Clinical trial design and aim of the clinical trial

**Figure 1:**
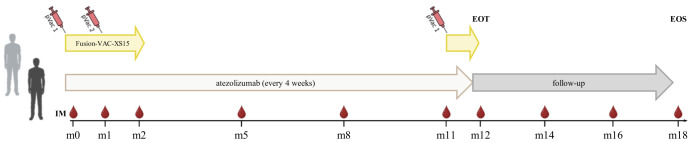
Treatment schedule of the FusionVAC22_01 clinical trial. Trial duration for each patient is approximately 1.5 years including 54 weeks of treatment and 6 months follow-up time. The vaccine Fusion-VAC-XS15 (500µl) will be administered subcutaneously (s.c.). Two vaccinations will take place with a 4-week interval at the beginning of the treatment phase. After 11 months a booster vaccination can be applied depending on T-cell responses. 1680mg atezolizumab will be applied every 4 weeks starting at day 15 after the first vaccination. Anti-PD-L1 treatment will be continued after end of vaccination phase until end of treatment phase or until disease progression or occurrence of a ≥ grade 3 adverse event that requires permanent discontinuation of Fusion-VAC-XS15 or atezolizumab or other reason for study termination. In case of temporary therapy interruptions, the planned therapy time may be extended to apply the planned maximum number of 13 atezolizumab doses. End of treatment (EOT) visit will be conducted 4 weeks after the 13^th^ and last atezolizumab application or in case of preliminary termination of the treatment 4 weeks after last administration of an investigational medicinal product (IMP). After end of treatment phase, patients will enter a 6-month follow-up period, including 2 follow-up visits taking place 2 and 4 months after last administration of an IMP. The final end of study visit (EOS) will be performed 6 months after last administration of an IMP. pVac, peptide vaccination with Fusion-VAC-XS15; IM, Immune monitoring; m, month. This figure was created with BioRender.com.

The FusionVAC22_01 trial is a phase-I, open-label, multi-center clinical trial designed to evaluate the safety, tolerability and preliminary efficacy of the DNAJB1-PRKACA fusion transcript-based peptide vaccine Fusion-VAC-XS15, in combination with anti-PD-L1 immune checkpoint inhibition with atezolizumab in patients with FL-HCC or other cancer entities carrying the DNAJB1-PRKACA fusion transcript. Trial duration for each patient will be approximately 1.5 years, including a treatment phase of up to 54 weeks and a 6 months follow-up period (**Figure 1**).

### 2.2 Selection of subjects and study population

The study population will comprise 20 patients with confirmed diagnosis of FL-HCC or other cancer entities carrying the DNAJB1-PRKACA fusion transcript fulfilling the inclusion criteria outlined below. Trial population will consist of both genders. Gender distribution in the trial is supposed to reflect the distribution in the real patient population and there will be no priorly defined quantitative ratio between females and males.

#### 2.2.1 Inclusion criteria

- Ability to understand and willingness to sign a written informed consent document.
- Histologically confirmed FL-HCC or other malignant disease that is locally advanced or metastatic.
- Presence of DNAJB1-PRKACA fusion transcript, assessed by ribonucleic acid (RNA)-based next generation sequencing (NGS) or reverse transcription polymerase chain reaction (RT-PCR).
- Non-FL-HCC patients can be included.
  ◦ in case of disease progression after therapy and fulfilling at least one of the following criteria.
    (i) no further standard therapy is available.
    (ii) patient is considered unsuitable for further available standard therapy.
    (iii) patient is unwilling to receive treatment with available standard therapy.
  ◦ if no standard therapy exists.
- Age ≥ 18 years.
- Eastern cooperative oncology group (ECOG) performance status 0 or 1.
- Patients must have measurable disease per response evaluation criteria in solid tumors modified for immunotherapies (iRECIST).
- Adequate organ function laboratory values.
  ◦ Absolute Lymphocyte Count > 500/µl.
  ◦ Platelets > 50.000/µl.
  ◦ Creatinine clearance glomerular filtration rate (GFR) > 30 ml/min.
  ◦ Liver function Child-Pugh index class A or B7.
  ◦ Alanine aminotransferase (ALT) and aspartase aminotransferase (AST) ≤ 5 times upper limit range.
  ◦ Bilirubin ≤ 3 mg/dl.
- Negative severe acute respiratory syndrome coronavirus 2 (SARS-CoV-2) rapid antigen test (as long as world health organization (WHO) declares pandemic spread of SARS-CoV-2).
- Negative serological hepatitis B test or negative polymerase chain reaction (PCR) test in case of positive serological test without evidence of an active infection, negative testing of hepatitis C RNA, negative human immunodeficiency virus (HIV) test within 6 weeks prior to study inclusion.
- Female patients of child bearing potential (FCBP) and male patients with partners of child bearing potential, who are sexually active, must agree to the use of two effective forms (at least one highly effective method) of contraception. This should be started from the signing of the informed consent and be continued until 5 months (both female and male patients) after last dose of an investigational medicinal product (IMP) (atezolizumab or vaccination).
- For FCBP, two negative pregnancy tests (sensitivity of at least 25 mIU/mL) prior to first application of a study drug (first vaccination), one at screening and the other one < 24h to first vaccination.
- Postmenopausal or evidence of non-child-bearing status.

#### 2.2.2 Exclusion criteria

- Pregnant or breastfeeding.
- Unwilling or unable to follow the study schedule for any reason.
- Chemotherapy or other systemic therapy or radiotherapy up to 14 days prior to the first dose of study drug.
- Concurrent or previous treatment within 30 days in another interventional clinical trial with an investigational anticancer therapy or any other investigational therapy, which would interfere with the study`s primary and secondary endpoints.
- Major surgery within 28 days of dosing of study drug.
- Have not recovered from adverse events (AEs) to grade ≤ 2 or baseline due to previous agents administered excluding alopecia and neurotoxicity (≤ 2 grade).
- History of autoimmune phenomena due to treatment with immunotherapy agents (including, anti-programmed cell death protein 1 (PD-1), anti-programmed cell death-ligand 1 (PD-L1), anti-programmed cell death-ligand 2 (PD-L2), anti-cytotoxic T-lymphocyte-associated protein 4 (CTLA4) antibodies, etc.) (≥ grade 3).
- Treatment with immunotherapy agents (including, anti-PD-1, anti-PD-L1, anti-PD-L2, anti-CTLA4 antibodies, etc.) within 28 days of dosing of study drug.
- Have received any live vaccine within 28 days prior to study treatment.
- Known sensitivity to or history of allergic reactions to any of the investigational drugs or known hypersensitivity to Chinese hamster ovary cell products.
- History of severe allergic anaphylactic reactions to chimeric, human or humanized antibodies, or fusion proteins.
- Active autoimmune disease that requires or has required systemic immunosuppressive treatment in the past 2 years.
- Presence of any tissue or organ allograft, regardless of need for immunosuppression, including corneal allograft. Patients with a history of allogeneic hematopoietic stem cell transplant will be excluded.
- Diagnosis of immunodeficiency.
- Systemic treatment with either corticosteroids (> 10mg daily prednisone equivalents) or other immunosuppressive medications within 7 days prior to study drug administration.
- Symptomatic interstitial lung disease.
- Active or untreated brain metastases or leptomeningeal metastases.
- Uncontrolled intercurrent illness including, but not limited to, uncontrolled infection, symptomatic congestive heart failure, unstable angina, cardiac arrhythmia, different metastatic cancer than the one leading to study enrollment or psychiatric illness/social situations that would limit compliance with study requirements.

### 2.3 Treatment of subjects

Trial duration for each patient will be approximately 1.5 years, including a treatment phase of up to 54 weeks and a 6 months follow-up time (**Figure 1**). During the treatment phase the two investigational medicinal products (IMPs) Fusion-VAC-XS15 and atezolizumab will be applied.

**Figure 2:**
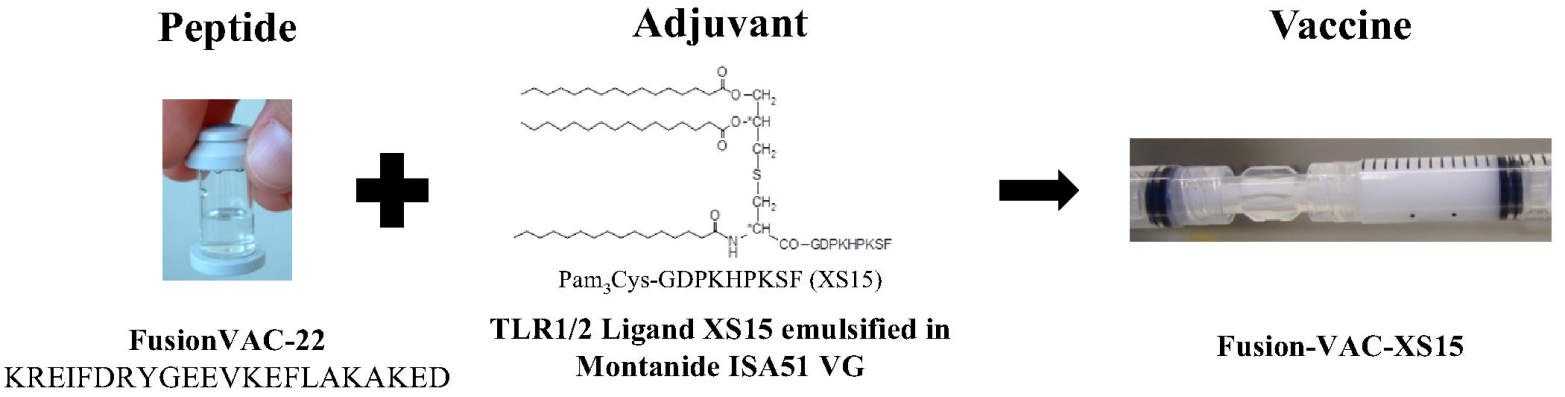
Vaccine composition. The DNAJB1-PRKACA fusion transcript-based peptide vaccine (Fusion-VAC-XS15) consists of 300µg of a 22mer neoepitope spanning the fusion region (FusionVAC-22) adjuvanted with the TLR 1/2 ligand XS15 (50µg) emulsified in Montanide ISA 51 VG (1:1).

The vaccine Fusion-VAC-XS15 (IMP, 500µl) consists of 300µg of a 22mer peptide neoepitope (HLA class II ligand KREIFDRYGEEVKEFLAKAKED) spanning the fusion region (FusionVAC-22) adjuvanted with the TLR 1/2 ligand XS15 (50µg) emulsified in Montanide ISA 51 VG (1:1) (**Figure 2**). Fusion-VAC-XS15 will be injected subcutaneously (s.c.) into the skin at the lower part of the abdomen of the patients. Emulsified XS15-adjuvanted vaccine peptides will persist at the vaccination side enabling a continuous immune stimulation resulting in the formation of a local granuloma (45, 46). Vaccination will take place at the start of the treatment phase. A total of two vaccinations are planned with a 4-week interval between them. After 11 months, a booster vaccination can be applied depending on T-cell responses (**Figure 1**).

1680mg atezolizumab (IMP), a humanized immunoglobulin G1 monoclonal antibody, that targets PD-L1, will be applied every 4 weeks as a 30-minute infusion (60-minute first dose) starting at day 15 after first vaccination. Anti-PD-L1 treatment will be continued after end of vaccination phase until end of treatment phase or until disease progression or occurrence of a ≥ grade 3 AE that requires permanent discontinuation of Fusion-VAC-XS15 or atezolizumab or other reason for study termination. In case of temporary therapy interruptions, the planned therapy time may be extended to apply the planned maximum number of 13 atezolizumab doses (**Figure 1**).

End of treatment (EOT) visit will be conducted 28 days (± 7 days) after the 13^th^ and last atezolizumab application or in case of preliminary termination of the treatment 28 days (± 7 days) after last administration of an IMP. After end of treatment phase, patients will enter a 6-month follow-up period, including 2 follow-up visits (FU) taking place 2 and 4 months (± 7 days) after last administration of an IMP. The final end of study visit (EOS) will be performed 6 months (± 7 days) after last administration of an IMP (**Figure 1**).

FCBP must have two negative pregnancy tests prior to first application of a study drug (first vaccination), one at screening and one at the first vaccination visit prior (< 24h) to first vaccination. FCBP must have one negative pregnancy test (sensitivity of at least 25 mIU/mL) prior (< 24h) to every application of an IMP (Fusion-VAC-XS15 or atezolizumab). The subject may not receive any IMP until the study doctor has verified that the results of these pregnancy tests are negative. In addition, pregnancy tests should be repeated throughout study treatment for FCBP every 28 days until 5 months after last dose of an IMP, at EOT visit, FU visits, at EOS visit and at study discontinuation.

Sexually active men and women of child-bearing potential must use two methods of reliable contraception including one highly effective (Pearl Index < 1) and one additional effective (barrier) method for up to 5 months (both female and male patients) after the last dose of an IMP (Fusion-VAC-XS15 or atezolizumab).

#### 2.3.1 Permanent termination of treatment with an IMP and further measures

Reasons for premature permanent termination of study drug treatment for an individual trial subject are:

- disease progression not requiring prompt alternative treatment.

- occurrence of a ≥ grade 3 AE that requires permanent discontinuation of IMPs.

If criteria for premature trial termination are not fulfilled (refer to section 2.3.2), patient will stay inside the study for safety reasons without receiving any IMP treatment. The patient is planned to attend all scheduled visits (after the time point of discontinuation of the IMP-treatment). Note, that the EOT visit must be conducted 28 days ± 2 days after last administration of an IMP. At investigators discretion some visits can be omitted, if not necessary for safety reasons.

#### 2.3.2 Premature termination of the clinical trial for a trial subject

Reasons for premature termination of trial for an individual trial subject are:

1. Death.
2. Withdrawal of consent.
3. Patient lost to follow-up.
4. Major protocol violation.
5. At their own request or at request of the legal representative.
6. Progressive disease requiring prompt alternative anti-cancer treatment. Treatment will be terminated, re-challenging with IMPs is not allowed. All patients will enter survival follow-up. In the unlikely event of progressive disease not requiring prompt alternative treatment, patient may stay on study for safety reasons (see also section 2.3.1).
7. Alternative anti-cancer treatment.
8. Occurrence of a ≥ grade 3 AE that requires permanent discontinuation of IMPs. If possible, patient will stay on study for safety reasons, but treatment will be terminated and re-challenging with IMPs is not allowed (see also section 2.3.1).
9. If, in the investigator’s opinion, continuation of the trial would be detrimental to the subject’s well-being.
10. For women, in case of pregnancy.
11. Non-compliance.
12. Non-compliance by the patient with protocol requirements.

All examinations scheduled for the last trial day will be performed and documented as far as possible, subject to the consent of the patient. Patients will enter the regular follow-up of the trial, unless consent to further study-related procedures has been withdrawn.

All ongoing AEs/SAEs of withdrawn subjects have to be followed-up until no more signs and symptoms are verifiable or the subject is on stable condition.

Premature termination should be avoided. In case of a premature termination of therapy, reasons/circumstances and if applicable the final status have to be documented. If the patient does not withdraw the consent for further follow-up, he/she should be followed-up as planned.

### 2.4 Study endpoints

#### 2.4.1 Primary endpoints

- Percentage of patients with induction of a peptide specific T-cell response until 28 days after second vaccination compared to baseline (prior to first vaccination) as determined by Interferon-gamma (IFNγ) enzyme-linked immunosorbent spot (ELISPOT).
- Incidence and severity of adverse events (AEs) including adverse events of special interest (AESIs), serious adverse events (SAEs) and suspected unexpected serious adverse reactions (SUSARs) (common toxicity criteria for adverse events (CTCAE) V5.0) until EOT visit or, in case of early termination, last assessment.

The induction of peptide-specific T-cell responses will be determined by IFNγ ELISPOT assays. Patients will be considered analyzable when they have received at least two vaccinations, and data on immunogenicity is available.

#### 2.4.2 Secondary endpoints

Immunogenicity:

- Percentage of patients with induction of a peptide specific T-cell response at indicated time points compared to baseline (prior to first vaccination) as determined by IFNγ ELISPOT until EOS visit.
- Number and percentage of patients receiving a booster vaccination out of all patients that are still under study treatment at the booster visit.

Safety:

- Incidence and severity of adverse events (AEs) including AESIs, SAEs and SUSARs (CTCAE V5.0) from first vaccination until EOS visit.

Efficacy, anti-tumor activity:

- Best objective tumor response assessed by iRECIST on routine imaging until end of study.
- Disease control rate (complete response (CR), partial response (PR), stable disease (SD)) until end of study.

Survival:

- Overall survival (OS) and Progression Free Survival (PFS) until end of study.

Quality of life:

- Overall quality of life scores throughout the study.

#### 2.4.3 Exploratory objectives

A correlation analysis of inducibility of immune responses with clinical and biological (including PD-L1 status and HLA allotyping) characteristics will be performed outside the study protocol. Also, an in-depth characterization of vaccine-induced T-cell responses will be conducted. Furthermore, differences in diverse cancer entities carrying the DNAJB1-PRKACA fusion transcript, e.g. regarding induction of immune response, safety and toxicity as well as efficacy of the peptide vaccine in combination with anti-PD-L1 immune checkpoint inhibition will be analyzed. In addition, for all patients participating in this study, PFS and OS will be assessed two and five years after the end of the study outside the study protocol.

#### 2.4.4 Assessment of efficacy

Immunological efficacy:

- Serial measurements of immunological efficacy will be performed on a regular basis at indicated time points throughout the clinical trial (**Figure 1**). Induction of peptide-specific CD8^+^ and CD4^+^ T cells will be evaluated, using IFNγ ELISpot (primary endpoint) at all indicated time points. In addition, intracellular cytokine staining for TNF and IFNγ, multi-color flow cytometry analysis for tetramer positive T cells, cytotoxicity analysis of peptide-specific T cells, and single cell mRNA sequencing will be performed to further analyze the immunological efficacy and if biopsy tissue will be available in single patients during the trial or at disease progression from tumor infiltrating lymphocytes.

Criteria for a booster vaccination:

- Less than 200 normalized spot counts (spot counts per 500.000 cells minus the respective negative control) assessed by IFNγ ELISPOT assay 7 months after second vaccination.
- > 50% decrease in intensity of T-cell response, normalized spot counts (spot counts per 500.000 cells minus the respective negative control) assessed by IFNγ ELISPOT assay 7 months after second vaccination compared to 28 days after second vaccination.

Tumor response by imaging iRECIST:

- Tumor assessment will be evaluated by computer tomography (CT) or magnetic resonance imaging (MRI) and progression or response will be based on iRECIST (47). Routine CT/MRI imaging has to be available at the time of study entry and be not older than 3 weeks. The choice of either CT or MRI depends on tumor localization and is conducted according to standard practice. During study treatment, imaging will be performed every 8 weeks, as routinely recommended (S3 Leitlinie: Diagnostik und Therapie des Hepatozellulären Karzinoms und biliärer Karzinome, Version 3.0 - Juli 2022, AWMF-Registernummer: 032/053OL) (48).

### 2.5 Safety

The safety and toxicity of the DNAJB1-PRKACA fusion transcript-based peptide vaccine in combination with anti-PD-L1 immune checkpoint inhibition will be determined based on the Common Terminology Criteria for Adverse Events (CTCAE V 5.0) and assessed in a descriptive manner. Serial measurements of safety will be performed at screening and at scheduled intervals throughout the study. All AEs and serious AEs will be documented and reported according to good clinical practice guidelines. Furthermore, we will report on AESIs, which include:

- Anaphylactic reactions (within 48h after application) to Fusion-VAC-XS15 administration.
- Cases of potential study drug-induced liver injury that include elevated ALT or AST in combination with either elevated bilirubin or clinical jaundice, as defined by Hy’s Law.
- Suspected transmission of an infectious agent by a study treatment, as defined below:
- Any organism, virus, or infectious particle (e.g., prion protein transmitting transmissible spongiform encephalopathy), pathogenic or non-pathogenic, is considered an infectious agent. A transmission of an infectious agent may be suspected from clinical symptoms or laboratory findings that indicate an infection in a patient exposed to a medicinal product. This term applies only when a contamination of the study treatment is suspected.
- Systemic lupus erythematosus.
- Events suggestive of hypersensitivity, infusion-related reactions, cytokine release syndrome (CRS), hemophagocytic lymphohistiocytosis (HLH), and macrophage activation syndrome (MAS).
- Nephritis.
- Ocular toxicities (e.g., uveitis, retinitis, optic neuritis).
- Grade ≥ 2 cardiac disorders.
- Vasculitis.
- Autoimmune hemolytic anemia.
- Severe cutaneous reactions (e.g., Stevens-Johnson syndrome, dermatitis bullous, toxic epidermal necrolysis).
- Myelitis.
- Facial paresis.

### 2.6 Data and safety monitoring board (DSMB)

An independent DSMB composed of independent experts in the field of oncology and immunology will assess the progress, safety data and critical efficacy endpoints. The DSMB will meet on a regular basis once a year. An emergency meeting of the DSMB may be called at any time should questions of patient safety arise (e. g. occurrence of vaccine related SAE), and necessary safety reports are provided. The DSMB will receive a report listing and summarizing all the relevant safety data once a year. In addition, the report provides data concerning recruitment rates and status of the trial. Based on its review, the DSMB provides the sponsor with recommendations regarding trial modification, continuation or termination of the trial. The DSMB also has to assess whether any stopping rule as defined per protocol is reached.

### 2.7 Sample size calculation

As usual in early phase I clinical trials, statistical planning is designed as such that a statistically reasoned decision for or against a subsequent phase II clinical trial can be made.

The sample size of the clinical trial was chosen based on the assumption that, in the case of peptide specific immune response induction in ≤ 30% of the patients, the therapy concept is extended with a probability of at most 5% (type one error, one-sided). On the other hand, in the case of peptide specific immune response induction in ≥ 60% of the patients, the therapy concept should be followed with a probability of at least 80% (power).

With a sample size of n = 20 patients, this means that at least 10 patients must have an immune response, so that the therapy concept is recommended for further evaluation in a randomized phase II study. The exact power is 87%, the exact type 1 error is 4.8% (calculations based on the binomial distribution with n = 20, p = 0.3 or p = 0.6, k < 10 or k ≥ 10).

To assure a patient number of n = 20 and thus enough statistical evidence for proceeding to a phase II trial, any subject that is excluded prior to second vaccination will be replaced. Patients after second vaccination will be replaced in case no data on immunogenicity are available.

## 3 Discussion

The FusionVAC22_01 trial is a phase I, open-label, multi-center clinical trial designed to evaluate the safety, tolerability and preliminary efficacy of a DNAJB1-PRKACA fusion transcript-based peptide vaccine Fusion-VAC-XS15, in combination with anti-PD-L1 immune checkpoint inhibition with the PD-L1 antibody atezolizumab in patients with FL-HCC or other cancer entities carrying the DNAJB1-PRKACA fusion transcript. Based on recent advances in genome sequence analysis (25, 26) that enabled the identification of further cancer entities comprising for example oncocytic neoplasms of the pancreas and bile duct that express the DNAJB1-PRKACA fusion transcript (27) a basket concept was applied for this trial to allow all tumor patients with metastatic disease and evidence of the DNAJB1-PRKACA fusion to participate. In view of the increasing personalization of tumor diagnostics and therapy, such basket concepts will allow the evaluation and subsequent drug approval independent of tumor entity. In particular, in view of technical advances in tumor genome sequencing including the analysis of fusion transcripts, which will become part of personalized routine diagnostics for all tumor patients at initial diagnosis in the next years, the evaluation and approval of new therapies based on genetic alterations independent of tumor entity is of central importance. This is evidenced by the approval of tyrosine kinase inhibitors for tumor patients with NTRK fusion positive tumors (49, 50).

Several peptide vaccination studies have reported promising results in solid tumors (28–31) and hematologic malignancies (32–35) in terms of *in vivo* immunogenicity, however so far lacking broad clinical responses. This might be due to several yet unmet prerequisites for clinically effective peptide vaccination including the selection of antigens, optimal adjuvants and combinatorial therapies. A major problem for the development of peptide-based vaccines, is the lack of suitable target structures represented by HLA class I- and HLA class II T-cell epitopes that show highly frequent and specific presentation on tumor cells and are recognized by the immune system (51). Neoepitopes arising from tumor-specific mutations have been identified as the primary drivers of anti-cancer T-cell responses induced by ICI and were therefore proposed as main candidates for future antigen-specific immunotherapies (52–54). In line response to ICI was shown to correlate with high tumor somatic mutational burden and some promising first results of neoepitope-based immunotherapies were observed in individual cancer patients (43, 55, 56). However, the broad application of this therapeutic approach is limited, especially in low-mutational burden cancer entities (57), due to the heterogeneity of somatic mutations among different tumor entities and affected individuals as well as the lack of a sufficient amount of somatic mutations that are ultimately presented as HLA-restricted neoepitopes on the tumor cells (52, 58–61). But in patients undergoing ICI treatment with low-mutational burden malignancies T-cell responses directed against gene fusion transcript-based neoepitopes were observed and correlated with treatment response (62). Such oncogenic gene fusion-derived neoepitopes have been proposed as a superior category of tumor antigens. This suggestion is based on (i) the clonal expression of oncogenic driver gene fusions (62, 63), (ii) the higher degree of sequence alteration compared to somatic point mutations, resulting in increased immunogenicity (64), and (iii) the limited ability of downregulation-based immune escape (65). However, only a very small part of *in silico* predicted neoantigens is actually naturally processed and presented via HLA molecules on the cancer cell (52, 58–61). This distorted relationship of gene expression and HLA-restricted presentation of the corresponding gene product is calling for direct methods of peptide target identification for vaccination approaches, which can be realized by mass spectrometry-based analysis of the entirety of naturally presented HLA ligands, termed the immunopeptidome of cancer cells (59, 66). Our extensive preclinical and first clinical findings identified the DNAJB1-PRKACA protein as source for immunogenic neoepitopes, and provided first efficacy data of T cell-based immunotherapy specifically targeting this oncogenic fusion (36). An off-the-shelf use of DNAJB1-PRKACA-based neoepitopes for immunotherapeutic cancer approaches is possible, because translation of the DNAJB1-PRKACA fusion results in a defined and unique protein in contrast to other fusion transcripts (36, 67–69). We validated the DNAJB1-PRKACA fusion protein as a source of HLA class I and HLA class II-presented antigens inducing both CD8^+^ and CD4^+^ T-cell responses, required for effective anti-cancer immunity (36, 70, 71). Furthermore, we proved the cellular processing and HLA-restricted presentation of these DNAJB1-PRKACA neoepitopes, which is an indispensable prerequisite for therapeutically used tumor antigens, in particular regarding the distorted correlation between gene expression and HLA-restricted antigen presentation (36, 52, 57, 72, 73).

Fusion-VAC-XS15 will be applied HLA allotype-independently in this trial, because the DNAJB1-PRKACA- based neoepitope peptide FusionVAC-22 is *in silico* predicted to bind to 1,290 different HLA class II alleles (36). Additionally, peptide sequences from 13 HLA of the 20 most frequent class I alleles are embedded within the sequence of FusionVac-22 (36). These 13 HLA class I alleles cover 96.6% and 93.8% of the European and world population with at least one HLA allotype, respectively (36). Nevertheless, in this trial HLA allotyping for all study patients is included, and within the exploratory objectives of the trial a correlation of induction of a T-cell response after vaccination to HLA status will be performed.

Besides the selection of optimal antigen targets, a further important prerequisite is the usage of suitable adjuvant drugs, that are able to induce strong and long-lasting immune responses. In this clinical trial we will use the TLR 1/2 agonist XS15, developed in Tübingen that is 1) water-soluble and 2) good manufacturing practice (GMP)-amenable, 3) non-toxic and 4) highly effective for inducing peptide-specific T cells *in vivo* (45, 46, 74). XS15-adjuvanted vaccines were evaluated in several trials in healthy individuals (NCT04546841), cancer patients with B cell/antibody deficiency (NCT04954469), glioblastoma patients (NCT04842513) and patients with chronic lymphocytic leukemia (NCT02802943). Here, peptide vaccines adjuvanted with the TLR1/2 ligand XS15 (50µg) emulsified 1:1 in Montanide ISA 51 VG were proven to be safe and effective to induce profound and long lasting CD4^+^ and CD8^+^ T cell responses that by far exceeded those induced by previous peptide vaccines as well as by mRNA-based vaccines (45, 46, 75, 76). In line with that, application of a DNAJB1-PRKACA fusion transcript-based peptide-vaccine adjuvanted with the TLR1/2 agonist XS15 emulsified in Montanide ISA 51 VG in one FL-HCC patient was well tolerated and showed the induction of profound and long-lasting T-cell responses accompanied by long-term disease-free survival (36).

Another important factor for the clinical effectiveness of peptide-based immunotherapy is the rational combination with other immunotherapies or cancer drugs. The induction (priming) of tumor-specific T-cell responses combined with an expansion of the immune response, and optimization of immune function within the tumor microenvironment achieved by immune checkpoint inhibitors has the potential to improve response rates and durability of responses in malignant diseases. The combination of vaccine approaches and immune checkpoint inhibitors is evaluated in multiple clinical trials for a variety of tumor entities including FL-HCC, showing promise as a means to facilitate the immune system and increase efficacy without substantially increasing toxicity (reviewed in (41–44)). Nevertheless, we implemented close monitoring of immunological side effects under the combination of peptide vaccines and ICI in the trial, in particular due to the possibility of severe or fatal courses of immune mediated side effects. This will comprise specific patient training for signs of immune-mediated adverse events, as well as close control of laboratory values. The sequence of peptide vaccination and ICIs, namely the application of peptide vaccine 15 days prior to the application of the first ICI dose is based on previous data from preclinical and clinical trials showing that after vaccination with neoantigens in patients, the expression of both PD-1 on neoantigen-specific T cells and PD-L1 in tumor tissues increases, and anti-PD-1 or anti-PD-L1 immunotherapy improves the efficacy of vaccines (41, 43). Peptide-based vaccination targeting tumor-specific, naturally presented antigen is a promising immunotherapeutic treatment to induce and augment T cell anti-tumor response in patients with lacking pre-existing tumor-reactive immune responses e.g. in low tumor mutational burden with insufficient response to immune checkpoint inhibition (ICI) alone (42, 43, 77–79). Vaccination approaches, enable the induction and expansion of tumor-specific T cell response far away from the tumor immune-suppressive microenvironment (6–9, 22–29). Moreover, cancer vaccines were shown to induce epitope spread further broadening the tumor-specific immune response and thus increasing the efficacy of ICIs in tumor entities responding modest or not at all to ICI-monotherapy (42). Additionally, antigen-specific T cells generated by cancer vaccines have been shown to express suppressor molecules to be targeted via ICI therapy to break this particular tumor resistance mechanism (80). In terms of FL-HCC and fusion-transcripts as targets for peptide vaccines, the combination with ICIs is further supported by (i) the recently reported observation that the immunosuppressive microenvironment of FL-HCC (40, 81) leading to T cell exclusion and exhaustion can be reversed via ICI and Il-10 blockade in fresh human FL-HCC tumor slice cultures (82) (ii) the high expression of PD-L1 in FL-HCC (81), (iii) case reports on FL-HCC patient treated successfully with ICIs (19, 37–39) as well as a multicenter retrospective analysis of a small number of FL-HCC patients showing a modest benefit from ICI monotherapy (40), and (iiii) the detection and response correlation of fusion protein-specific T cells in patients receiving ICIs (62).

Thus, the advantages of the combination of peptide vaccines and ICIs outweigh the potential risks of increased immune-mediated side effects, in particular with regard to the multiple risk mitigation procedures implemented in the clinical trial.

In this phase I clinical trial FL-HCC patients and other cancer patients with proof of the DNAJB1-PRKACA fusion will be treated to evaluate the immunogenicity along with safety and toxicity as well as first efficacy of the DNAJB1-PRKACA fusion transcript-based peptide vaccine Fusion-VAC-XS15 combined with the immune checkpoint inhibitor atezolizumab. The sample size of 20 patients is based on a sufficient T-cell induction rate as mandatory prerequisite for further evaluation of the treatment in a following randomized phase II study. Accordingly, primary objectives of this phase I clinical trial are (i) to assess immunogenicity in terms of induction of peptide specific T-cell responses and (ii) to assess safety and toxicity of the peptide vaccine Fusion-VAC-XS15 in combination with the anti-PD-L1 immune checkpoint inhibitor atezolizumab. To gain first evidence regarding efficacy best objective tumor response based on iRECIST on routine imaging, disease control rate (CR, PR, SD) and survival (OS, PFS) will be assessed as secondary objectives. Vaccine-induced T cell phenotypes and functionality as well as corresponding T cell receptor sequences will be comprehensively assessed using multi-color flow cytometry as well as single cell mRNA sequencing of vaccine induced T cells and, if biopsy tissue will be available in single patients during the trial or at disease progression, from tumor infiltrating lymphocytes. Also, the correlation of T-cell response with HLA allotyping (high resolution, four digits, for HLA class I A, B and C and HLA-DR, DQ, DP) and PD-L1 status, based on immunohistochemical analysis of tumor sections, will be assessed. Evaluation of the clinical trial results with regard to these objectives after end of the clinical trial, including safety, immunogenicity and first efficacy data of the treatment, will be performed to decide on implementation of a subsequent phase II clinical trial.

Taken together, the DNAJB1-PRKACA fusion transcript-based peptide vaccine Fusion-VAC-XS15 combined with the immune checkpoint inhibitor atezolizumab is an innovative and promising combinatorial immunotherapeutic treatment option for the devastating tumor disease FL-HCC and other cancer entities carrying the DNAJB1-PRKACA fusion transcript.

## Ethics statement

The studies involving humans were approved by Ethics Committee II of the University of Heidelberg (Medical faculty of Mannheim). The studies were conducted in accordance with the local legislation and institutional requirements. The participants provided their written informed consent to participate in this study.

## Author contributions

CH: Writing – review & editing, Writing – original draft, Visualization, Project administration, Investigation, Formal analysis, Conceptualization. JB: Writing – review & editing, Writing – original draft, Visualization, Investigation, Formal analysis. JH: Writing – review & editing, Writing – original draft, Visualization, Investigation, Formal analysis, Conceptualization. YM: Writing – review & editing, Writing – original draft, Investigation, Formal analysis. AN: Writing – review & editing, Writing – original draft, Investigation, Formal analysis. MD: Writing – review & editing, Writing – original draft, Resources, Investigation. LZ: Writing – review & editing, Writing – original draft, Resources, Investigation. CK: Writing – review & editing, Writing – original draft, Resources, Investigation. BF: Writing – review & editing, Writing – original draft, Investigation. SJ: Writing – review & editing, Writing – original draft, Investigation. PM: Writing – review & editing, Writing – original draft, Formal analysis, Data curation, Conceptualization. NM: Investigation, Writing – review & editing, Writing – original draft, Resources. KN: Resources, Writing – review & editing, Writing – original draft, Investigation, Formal analysis. HS: Writing – review & editing, Writing – original draft, Resources, Investigation, Formal analysis, Conceptualization. MB: Supervision, Writing – review & editing, Writing – original draft, Investigation, Funding acquisition, Conceptualization. JW: Writing – review & editing, Writing – original draft, Supervision, Resources, Project administration, Funding acquisition, Formal analysis, Conceptualization.
